# Role of LL-37 in thrombotic complications in patients with COVID-19

**DOI:** 10.1007/s00018-022-04309-y

**Published:** 2022-05-21

**Authors:** Zilei Duan, Juan Zhang, Xue Chen, Ming Liu, Hongwen Zhao, Lin Jin, Zhiye Zhang, Ning Luan, Ping Meng, Jing Wang, Zhaoxia Tan, Yaxiong Li, Guohong Deng, Ren Lai

**Affiliations:** 1 Southern Marine Science and Engineering Guangdong Laboratory (Guangzhou), Guangzhou, 511458 China; 2 Present Address: Key Laboratory of Animal Models and Human Disease Mechanisms of Chinese Academy of Sciences/Key Laboratory of Bioactive Peptides of Yunnan Province, KIZ-CUHK Joint Laboratory of Bioresources and Molecular Research in Common Diseases, Sino-African Joint Research Center, Center for Biosafety Mega-Science, Kunming Institute of Zoology, Kunming, 650223 Yunnan China; 3 Southwest Hospital, Third Military Medical University (Army Medical University, 29 Gaotanyan Street, Shapingba, Chongqing, 400038 China; 4 Department of Cardiovascular Surgery, Yan’an Affiliated Hospital of Kunming Medical University, Kunming, 650041 Yunnan China; 5 Department of Laboratory Diagnosis, Chongqing Public Health Medical Center, Public Health Hospital of Southwest University, 109 Baoyu Rd. Shapingba, Chongqing, 400038 China

**Keywords:** LL-37, SARS-CoV-2, Hypercoagulation, Coagulation factors, COVID-19

## Abstract

Blood clot formation induced by dysfunctional coagulation is a frequent complication of coronavirus disease 2019 (COVID-19) and a high-risk factor for severe illness and death. Neutrophil extracellular traps (NETs) are implicated in COVID-19-induced immunothrombosis. Furthermore, human cathelicidin, a NET component, can perturb the interaction between the SARS-CoV-2 spike protein and its ACE2 receptor, which mediates viral entry into cells. At present, however, the levels of cathelicidin antimicrobial peptides after SARS-CoV-2 infection and their role in COVID-19 thrombosis formation remain unclear. In the current study, we analyzed coagulation function and found a decrease in thrombin time but an increase in fibrinogen level, prothrombin time, and activated partial thromboplastin time in COVID-19 patients. In addition, the cathelicidin antimicrobial peptide LL-37 was upregulated by the spike protein and significantly elevated in the plasma of patients. Furthermore, LL-37 levels were negatively correlated with thrombin time but positively correlated with fibrinogen level. In addition to platelet activation, cathelicidin peptides enhanced the activity of coagulation factors, such as factor Xa (FXa) and thrombin, which may induce hypercoagulation in diseases with high cathelicidin peptide levels. Injection of cathelicidin peptides promoted the formation of thrombosis, whereas deletion of cathelicidin inhibited thrombosis in vivo. These results suggest that cathelicidin antimicrobial peptide LL-37 is elevated during SARS-CoV-2 infection, which may induce hypercoagulation in COVID-19 patients by activating coagulation factors.

**Supplementary Information:**

The online version contains supplementary material available at 10.1007/s00018-022-04309-y.

## Introduction

SARS-CoV-2 is the causative pathogen of coronavirus disease 2019 (COVID-19), a potentially life-threatening multi-organ disease that continues to impact the human population on a global scale. Accumulating evidence indicates that frequent thromboembolic complications affecting the venous and arterial vascular systems are an important risk factor in patients with COVID-19 [1–10]. Vascular complications caused by dysfunctional coagulation include venous thromboembolism (VTE), a composite of deep vein thrombosis (DVT) and pulmonary embolism (PE) [11–16]. Current evidence suggests that the incidence of thrombotic complications in COVID-19 patients admitted to intensive care units (ICUs) is between 16 and 49% [6–8, 10–12, 15]. Small thrombi have also been observed in the pulmonary arterioles of COVID-19 patients during autopsy [17, 18]. Basic characteristics of hypercoagulation, including d-dimers and fibrinogen elevation, platelet activation, and platelet-monocyte aggregate formation, are also found in critically ill COVID-19 patients, and is known as a coagulation storm [6–8, 16, 19–23]. The prophylactic use of low-molecular weight heparin (LMWH) is recommended in all hospitalized patients for anti-thrombosis, unless contraindicated [1, 11, 20]. Despite the presence of hypercoagulation, unexplained prolongation of activated partial thromboplastin time (APTT) and prothrombin time (PT) has also been reported in COVID-19 patients [24, 25]. At present, however, the factors that induce the above hematological findings in COVID-19 remain elusive.

Circulating markers of neutrophil extracellular traps (NETs), such as nucleosomal citrullinated histone H3 (H3Cit-DNA), cell-free DNA (cfDNA), and neutrophil elastase (NE), are increased in COVID-19 patients and NETs are known to contribute to immunothrombosis in COVID-19 acute respiratory distress syndrome [26–28]. Cathelicidin antimicrobial peptides, components of NETs [29], are reported to perturb the interaction between SARS-CoV-2 spike protein and its ACE2 receptor [30], which may inhibit viral infection. However, the levels of cathelicidin antimicrobial peptides and their role in the thrombosis formation in COVID-19 patients remain unclear.

Cathelicidins, which belong to the family of host defense peptides, play an important role in innate immunity [31]. They exhibit a broad-spectrum effect against pathogens via direct microbicidal and immunomodulatory activities [32]. LL-37 is the only human member of the cathelicidin antimicrobial peptide family and is derived from human cathelicidin antimicrobial protein 18 (hCAP18) by the cleavage of proteinase 3 [33]. Although cathelicidins have mostly been studied with respect to their antibacterial and immunomodulatory activity, elevated levels are reported to aggravate diseases, such as psoriasis [34], atherosclerosis [35] and ulcerative colitis [36], by induction of inflammation. Cathelicidins may initiate and propagate thrombosis by activating platelets [37, 38]; however, their role in coagulation cascade activation remains unclear.

Here, we investigated the role of LL-37 in COVID-19 patient coagulopathy. Results showed that increased LL-37 was correlated with COVID-19-related coagulation dysfunction. LL-37 may potentiate the activity of coagulation factors, such as FXa and thrombin, thereby contributing to hypercoagulation in COVID-19.

## Methods

### Experimental ethics

All human specimens and clinical information were collected with informed consent of the patients prior to the study from the Chongqing Public Health Medical Center (CPHMC) and Department of Infectious Diseases, Southwest Hospital, Third Military Medical University (Army Medial University). Patients with laboratory-confirmed COVID-19 (*n* = 62) and age- and sex-matched healthy controls (HCs, *n* = 21) were included in this study. For the measurement of LL-37, thrombin time (TT), fibrinogen, prothrombin time (PT) and activated partial thromboplastin time (APTT), numbers of each group indicated in the text. COVID-19 patients were divided into mild or moderate (MM, *n* = 40) and severe or critical (SC, *n* = 22) groups according to the Chinese Clinical Guidance for COVID-19 Pneumonia Diagnosis and Treatment (6th edition). Briefly, patients with mild or moderate (MM) disease were defined based on the following clinical symptoms: (1) Mild clinical symptoms, with no sign of pneumonia on chest imaging; (2) Fever and respiratory symptoms, with signs of pneumonia through radiological assessment. Patients with severe or critical (SC) disease were defined based on the following clinical symptoms: (1) Shortness of breath, respiratory rate (RR) ≥ 30 times/min, oxygen saturation ≤ 93% at rest, alveolar oxygen partial pressure/fraction of inspiration O_2_ (PaO_2_/FiO_2_) ≤ 300 mmHg; (2) Respiratory failure requiring mechanical ventilation, shock, combined with other organ failure needed ICU monitoring and treatment. Determination of LL-37 in the plasma of COVID-19 patients and HCs was approved by the Ethics Committee of Chongqing Public Health Medical Center (2020-002-01-KY, 2020-003-01-KY). The study and all animal experiments were approved by the Institutional Review Board and Animal Care and Use Committee at Kunming Institute of Zoology (SMKX-20201021-15).

### Reagents

Reagents were purchased from Sigma-Aldrich or indicated suppliers. Anti-β-actin mouse (sc-47778) and anti-LL-37 mouse monoclonal antibodies (sc-166770) were purchased from Santa Cruz Biotechnology. Anti-cathelicidin rabbit monoclonal antibodies (ab207758) were purchased from Abcam. Peroxidase-AffiniPure goat anti-rabbit IgG (111-035-003), peroxidase-AffiniPure goat anti-mouse IgG (115-035-003), and fluorescein (FITC)-AffiniPure donkey anti-mouse IgG (715-095-151) were purchased from Jackson ImmunoResearch Laboratories. The SARS-CoV-2 spike protein (Z03481) was purchased from GenScript. Human alpha thrombin (HT 1002a) and human factor Xa (HFXa 1011) were purchased from Enzyme Research Laboratories.

### Peptide synthesis

Peptides (LL-37: LLGDFFRKSKEKIGKEFKRIVQRIKDFLRNLVPRTES; Cramp: GLLRKGGEKIGEKLKKIGQKIKNFFQKLVPQPE; and FITC-labeled LL37 or FITC-labeled Cramp) were synthesized by GL Biochem (Shanghai, China) and their purities (> 98%) were confirmed by reversed phase high-performance liquid chromatography (RP-HPLC) and mass spectrometry.

### Mice

Male C57BL/6 J mice aged 6–8 weeks were purchased from Beijing HFK Bio-Technology Co. Ltd. (Beijing, China). Cramp knockout mice (*Cramp*^*−/−*^) were purchased from the Jackson Laboratory.

### Cell culture and treatment

The human lung epithelial cell line A549 was purchased from the Kunming Cell Bank and maintained in complete Dulbecco’s Modified Eagle Medium (Corning, 10-013-CVR) supplemented with 10% fetal bovine serum, 100 U/ml penicillin, and 100 μg/ml streptomycin (Gibco BRL, Gaithersburg, MD, USA) at 37 °C in 5% CO_2_.

To determine the effects of SARS-CoV-2 infection on LL-37 expression, A549 cells were stimulated with SARS-CoV-2 (MOI: 0.01, 0.05, 0.25) for 2 h at the biosafety level-3 laboratory of the Kunming High-level Biosafety Primate Research Center, Yunnan, China, with LL-37 expression then detected using confocal microscopy and enzyme linked immunosorbent assay (ELISA) after 24 h. To confirm whether the effects of SARS-CoV-2 on LL-37 expression were dependent on the spike protein, we stimulated A549 cells with spike protein (0.4–10 μg/ml), bovine serum albumin (BSA, 10 μg/ml, negative control), lipopolysaccharides (LPS, positive control) from *Escherichia coli* O111:B4 (10 μg/ml) for 24 h, then measured LL-37 expression using confocal microscopy and Western blot analysis.

### ELISA

The levels of LL-37 in the plasma of COVID-19 patients and supernatant of SARS-CoV-2 stimulated A549 cells were analyzed using a LL-37 ELISA kit (Hycult Biotech, HK321-01) according to the manufacturer’s instructions. A binding assay between cardiolipin and LL-37 was carried out by ELISA according to previously described methods [39]. Briefly, a 96-well white plate (Corning, Kennebunk ME, USA) was filled with 50 μl of 50 μg/ml cardiolipin diluted in ethanol and evaporated at 4 °C. After washing with phosphate-buffered saline (PBS; pH 7.4), the wells were blocked with 2% BSA in PBS (1 h at room temperature). Then, 100 μl of FITC-labeled LL-37 or FITC-labeled Cramp (10 μg/ml) was added, followed by incubation for 1 h at 37 °C. After washing with PBS (pH 7.4), fluorescence was detected using a Cytation 3 Cell Imaging Multi-Mode Reader (Biotek), and the binding of LL-37 with cardiolipin was calculated. In all assays, ethanol-treated wells were used as negative controls.

### Western blot analysis

After stimulation with the SARS-CoV-2 spike protein (0.4–10 μg/ml) for 24 h, A549 cells were homogenized and sonicated in RIPA buffer (Sigma: 150 mM NaCl, 1.0% IGEPAL^®^ CA-630, 0.5% sodium deoxycholate, 0.1% sodium dodecyl sulfate (SDS), 50 mM Tris, pH 8.0). Insoluble material was removed by centrifugation (12 000 rpm, 4 ℃, 15 min). The cell homogenates were separated by tricine SDS–polyacrylamide gel electrophoresis (tricine-SDS-PAGE), then transferred to polyvinylidene difluoride (PVDF) membranes (Rainin, 0.22 μm). Anti-cathelicidin (ab207758) and anti-β-actin antibodies were used to detect cathelicidin and β-actin according to the manufacturer’s instructions.

### Coagulation functional assay and enzymatic activity assay of coagulation factors

Coagulation function assays (TT, APTT, PT) were conducted by detecting absorbance at 650 nm using the SUNBIO kit according to the manufacturer’s instructions. Enzymatic activity of coagulation factors was measured using chromogenic substrate. Briefly, coagulation factors (FXa, 0.1 nM, thrombin, 10 nM) and different amounts of peptides (final concentrations ranging from 0.0 to 16.2 μg/ml) were pre-incubated for 10 min at 37 °C. After incubation, the reaction was initiated by the addition of 0.5 mM substrate F3301 (CH3OCO-D-CHA-Gly-Arg-pNA-AcOH, Sigma) for FXa and H-D-Phe-Pip-Arg-pNa·2HCl (Hyphen Biomed, Neuville-sur-Oise, France) for thrombin, with the reaction monitored continuously at 405 nm.

To confirm the effects of cathelicidins on coagulation factors, we determined the role of LL-37 and Cramp on enzymatic activity of thrombin and FXa to their physiological substrates (fibrinogen and prothrombin, respectively). Briefly, LL-37 (0–50 μg/ml) or Cramp (0–50 μg/ml) was incubated with thrombin (5 nM in buffer: 100 mM NaCl, 50 mM Tris, 5 mM CaCl_2_, pH 8.0) and FXa (200 nM, in buffer: 100 mM NaCl, 50 mM Tris, 5 mM CaCl_2_, pH 8.0) for 10 min at 37 °C, respectively. Their physiological substrates, i.e., fibrinogen (2 μM) and prothrombin (10 μM), were added and incubated for another 10, 30 min at 37 °C, respectively. After that, reduced loading buffer was added and boiled for 5 min, and the samples were analyzed by SDS-PAGE and stained using Coomassie Blue.

To determine the enzymatic activity of thrombin and FXa in plasma from Cramp knockout (*Cramp*^*−/−*^) and C57BL/6 J mice (*n* = 6–7 per group), thrombin and FXa chromogenic substrates were added to the plasma, with the reaction initiated immediately and monitored continuously at 405 nm.

### Surface plasmon resonance (SPR) analysis

SPR analysis was performed as described previously, with some modifications [40]. Briefly, thrombin and FXa were immobilized on the activated sensor chip CM-5 by amine coupling. LL-37 or Cramp in HBS-EP + running buffer was applied to the immobilized ligand at a flow rate of 30 μl/min and the real-time binding signal was recorded using BIAcore 3000 (GE, USA). The equilibrium dissociation constant (*K*_D_) was calculated using the Langmuir model with Biacore evaluation software provided by the manufacturer.

### ***FeCl***_***3***_***-induced mouse thrombosis model***

Male C57BL/6 mice with or without cathelicidin antimicrobial peptides administration and *Cramp*^*−/−*^ mice (6–8 weeks old, *n *= 3–7 per group) were anesthetized with 2% isoflurane. The left carotid artery was exposed and visualized through a dissecting microscope. A Doppler microvascular probe (RWD Life Science, Shenzhen, China) was placed on the exposed artery to measure vascular blood flow. Thrombosis was induced by directly placing a small piece of filter paper saturated with 20% FeCl_3_ on the artery for 1.5 min. Time to vessel occlusion was measured when blood flow was completely stopped.

### *Acute pulmonary thromboembolism in *mice

LL-37 (100 μl, 30 mg/kg) or Cramp (100 μl, 30 mg/kg) in saline was injected into male C57BL/6 mice (6–8 weeks old, *n* = 5 per group) via the caudal vein. Control mice received the same volume of saline. At 10 min after the injection, the mice were anesthetized with pentobarbital sodium (50 mg/kg, intraperitoneal injection). The chest cavity was then exposed, and the right side of the heart was perfused with saline to remove blood from pulmonary circulation. After perfusion, the whole lung was excised and fixed in 4% paraformaldehyde dissolved in PBS at 4 °C overnight for histopathological examination.

### Confocal microscopy

For immunostaining, cells were fixed in 4% paraformaldehyde and PBS at 4 °C for 30 min and permeabilized with 0.1% Triton X-100 in PBS for 15 min before being treated with 2% BSA for 1 h at 25 °C. To analyze LL-37 expression in A549 cells, samples were incubated with anti-LL-37 mouse monoclonal antibodies (sc-166770). DAPI (1 μg/ml, Roche Diagnostics) was used to stain DNA. After washing with PBS, cells were incubated for 1 h at 37 °C with fluorescent-labeled fluorescein (FITC)-AffiniPure donkey anti-mouse IgG secondary antibodies (715-095-151). Cells were imaged using an Olympus FluoView 1000 confocal microscope (Olympus, Melville, NY, USA).

### Statistical evaluation

Data obtained from independent experiments were presented as mean ± standard deviation (SD). For normal continuous variables, one-way analysis of variance (ANOVA) was used. Comparisons of more than two groups were performed using Kruskal–Wallis one-way ANOVA followed by Dunn’s multiple comparison test using GraphPad Prism v5. Differences were considered significant at *p* < 0.05.

## Results

### Coagulation function in COVID-19 patients

**Fig. 1:**
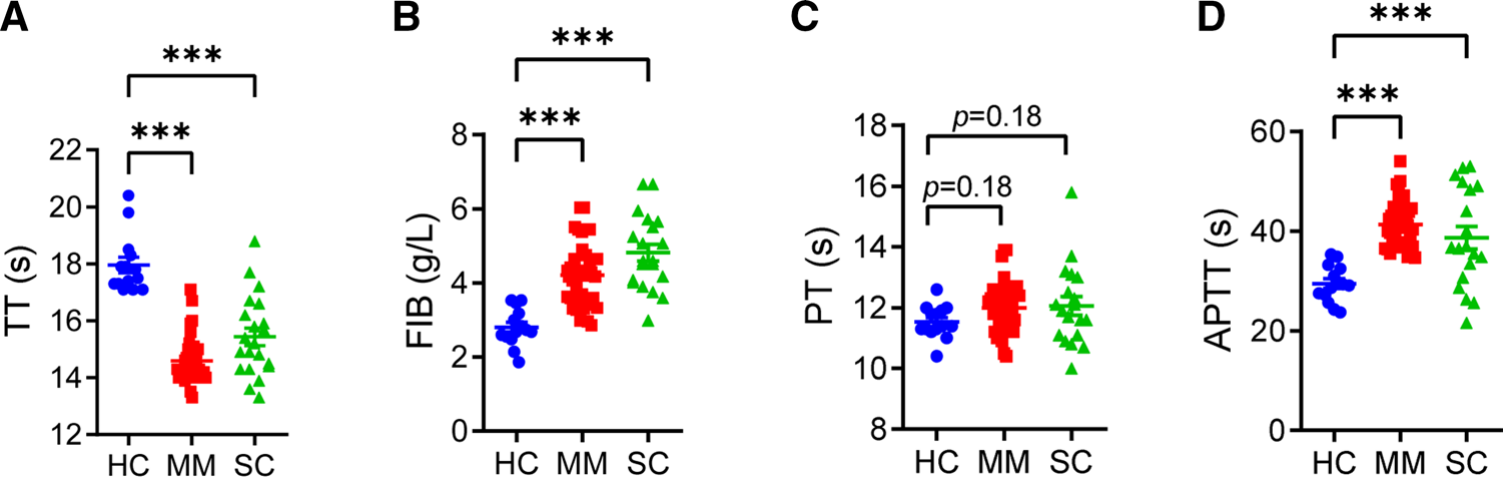
Coagulation function in COVID-19 patients. **A** Thrombin time (TT) in COVID-19 patients (mild and moderate (MM, *n* = 38), severe and critical (SC, *n *= 21) patients) and healthy controls (HCs, *n *= 14) was analyzed. **B** Fibrinogen (FIB) level in COVID-19 patients (MM, *n* = 40 and SC, *n* = 20) and HCs (*n *= 14) was analyzed. **C** Prothrombin time (PT) in COVID-19 patients (MM, *n* = 40 and SC, *n* = 19) and HCs (*n* = 14) was analyzed. **D** Activated partial thromboplastin time (APTT) in COVID-19 patients (MM, *n* = 40 and SC, *n* = 19) and HCs (*n* = 14) was analyzed. Data are mean ± SD of at least three independent experiments. *p < 0.05, **p < 0.01, ***p < 0.001

To assess coagulation function in COVID-19 patients, coagulation testing (TT, PT, APTT, fibrinogen) was performed. As illustrated in Fig. 1A, B, TT (HCs vs MM vs SC: 17.96 ± 0.98 s vs 14.60 ± 0.79 s vs 15.43 ± 1.37 s) was shortened and fibrinogen (HCs vs MM vs SC: 2.81 ± 0.48 g/L vs 4.21 ± 0.83 g/L vs 4.81 ± 0.98 g/L) was increased in COVID-19 patients compared to HCs. Both MM and SC patients showed significantly shortened TT and increased fibrinogen, suggesting hypercoagulation in COVID-19 patients. However, PT showed no significant difference between COVID-19 patients (MM: 12.00 ± 0.80 s, SC: 12.06 ± 1.29 s) and HCs (11.53 ± 0.52 s) (Fig. 1C), while APTT (Fig. 1D) was elevated in COVID-19 patients (MM: 41.35 ± 4.48 s and SC: 38.70 ± 9.63 s) compared to HCs (29.44 ± 3.52 s), which are contradictory to the shortened TT and increased fibrinogen level. These results suggest that certain factors exist in the blood of COVID-19 patients, which could affect PT and APTT.

### Elevated LL-37 in plasma of COVID-19 patients

**Fig. 2:**
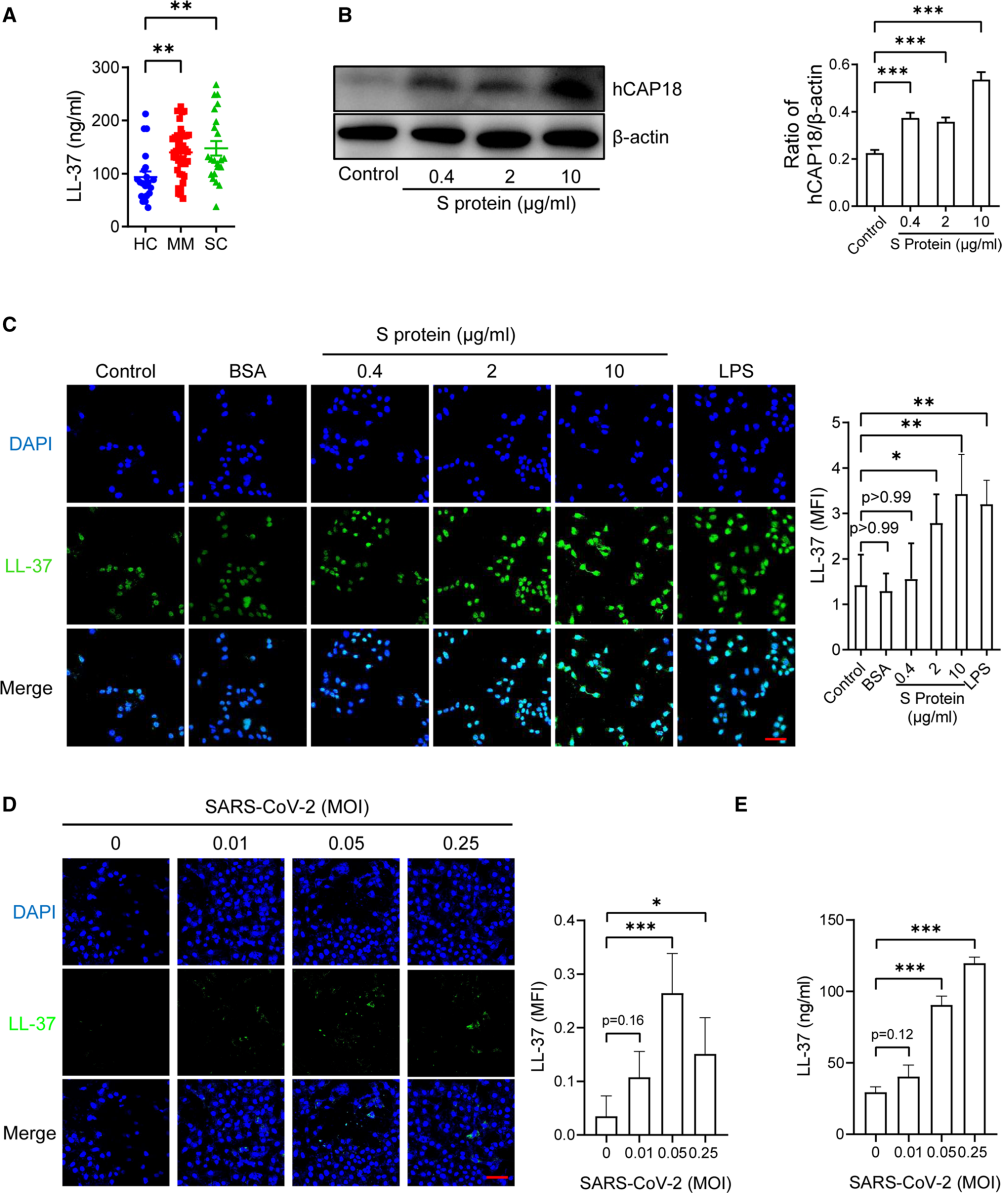
SARS-CoV-2 spike protein upregulates LL-37 expression. **A** Amount of LL-37 in plasma of COVID-19 patients (MM, *n* = 38 and SC, *n* = 22) and HCs (*n *= 21) was detected by ELISA. **B** hCAP18 expression in A549 cells with spike protein (0.4, 2, 10 μg/ml) stimulation for 24 h was detected by Western blot analysis. Representative images of Western blots (left) and quantification of the hCAP18 expression (ratio of hCAP18/β-actin, right). **C** LL-37 expression in A549 cells with spike protein (0.4, 2, 10 μg/ml), bovine serum albumin (BSA, 10 μg/ml, negative control), lipopolysaccharides (LPS, positive control) from Escherichia coli O111:B4 (10 μg/ml) stimulation for 24 h was detected by confocal microscopy. Representative images (left) and quantification results of LL-37 expression (right). (**D**) LL-37 expression in A549 cells with SARS-CoV-2 (MOI: 0.01, 0.05, 0.25) stimulation was determined by confocal microscopy. The representative images (left) and the quantification results of the LL-37 expression (right). Scale bar: 50 μm. Data are mean ± SD of at least three independent experiments. **p < 0.01, ***p < 0.001

Cathelicidin peptides increase during viral infection, which induces the formation of thrombosis by activating platelets [37, 38]. The human cathelicidin antimicrobial peptide LL-37 is reported to inhibit SARS-CoV-2 infection [30]. To investigate the role of cathelicidin peptides in SARS-CoV-2 infection, the level of LL-37 in the plasma of COVID-19 patients (MM and SC) and HCs was measured. As illustrated in Fig. 2A, the concentration of LL-37 in the plasma of COVID-19 patients (140 ± 46.47 ng/ml in MM and 147.6 ± 64.24 ng/ml in SC) was significantly higher than that in HCs (93.62 ± 48.14 ng/ml).

### SARS-CoV-2 upregulates LL-37 expression through the spike protein

To further investigate the association between LL-37 overexpression and SARS-CoV-2 infection, the expression of hCAP18 (precursor of LL-37) in A549 cells after SARS-CoV-2 spike protein (0.4–10 μg/ml) incubation for 24 h at 37 ℃ was determined by Western blot analysis. From the results of Fig. 2B, hCAP18 expression in A549 cells significantly increased after incubation with the spike protein. To further confirm the effects of the spike protein on LL-37 expression, we stimulated A549 cells with spike protein (0.4–10 μg/ml), BSA (10 μg/ml, negative control), LPS (positive control) from *Escherichia coli* O111:B4 (10 μg/ml) for 24 h, then measured LL-37 expression by confocal microscopy. As illustrated in Fig. 2C, BSA had no effect on LL-37 expression, whereas the spike protein and LPS significantly promoted LL-37 expression. Furthermore, to confirm the effects of SARS-CoV-2 infection on LL-37 expression, we stimulated A549 cells with SARS-CoV-2 (MOI: 0.01, 0.05, 0.25) for 2 h, then detected LL-37 expression using confocal microscopy and ELISA after 24 h. As illustrated in Fig. 2D, E, SARS-CoV-2 infection significantly elevated LL-37 expression. Thus, these results suggest that SARS-CoV-2 infection induced LL-37 expression through the spike protein.

### LL-37 is correlated with coagulation function in COVID-19 patients

**Fig. 3:**
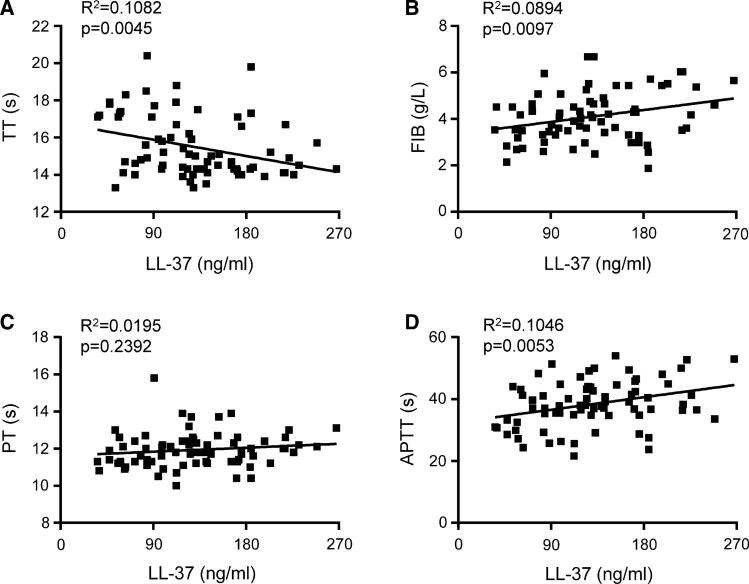
Analysis of correlation between LL-37 level and coagulation function. Correlation analysis of LL-37 level and TT (**A**), fibrinogen (**B**), PT (**C**), and APTT (**D**)

As LL-37 expression and coagulation function changed in COVID-19 patients, we investigated the correlations between LL-37 and TT, fibrinogen, PT, and APTT. As shown in Fig. 3A–D, LL-37 was negatively correlated with TT (Fig. 3A, *R*^2^ = 0.1082, *p* = 0.0045) and positively correlated with fibrinogen level (Fig. 3B, *R*^2^ = 0.0894, *p* = 0.0097) and APTT (Fig. 3D, *R*^2^ = 0.1046, *p* = 0.0053), but showed no correlation with PT (Fig. 3C, *R*^2^ = 0.0195, *p* = 0.2392).

### LL-37 promotes thrombosis formation through potentiation of coagulation factor activity

**Fig. 4:**
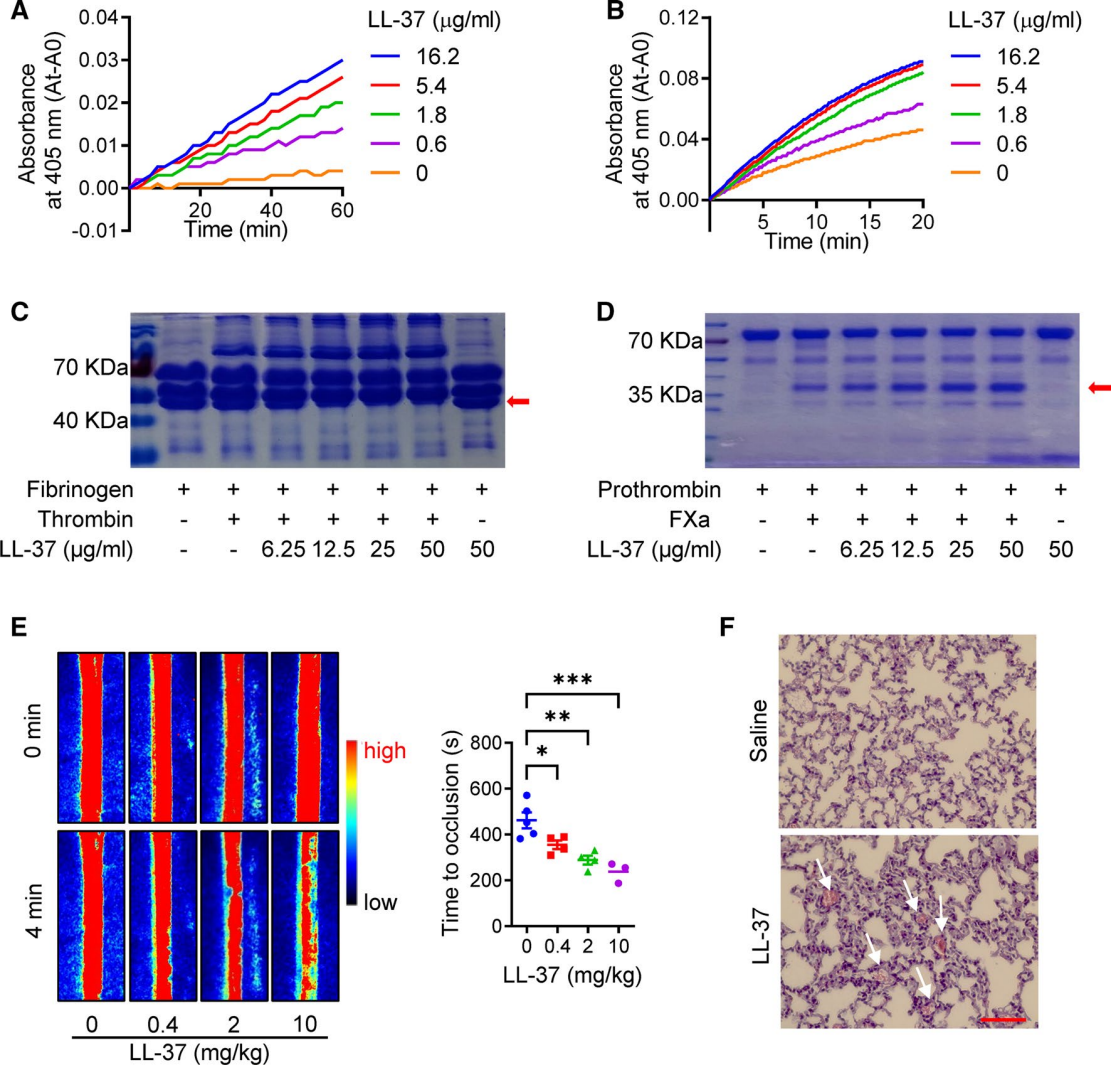
Promotion of LL-37 on thrombosis formation through potentiation of coagulation factor activity. LL-37 enhanced activity of thrombin (**A**, **C**) and FXa (**B**, **D**) on their chromogenic substrates (**A**, **B**) and physiological substrates (**C**, **D**). Red arrows in **C** and **D** indicated fibrin and thrombin, respectively. (**E**) LL-37 administration (0.4–10 mg/kg) induced thrombosis formation and shortened time of arterial occlusion in FeCl_3_-induced carotid artery thrombosis mouse model. Representative images of carotid artery blood flow at 0 and 4 min (left), and statistical analysis of vascular occlusion time (right) of each group (blue, 0 mg/kg; red, 0.4 mg/kg; green, 2 mg/kg, purple, 10 mg/kg). (**F**) LL-37 (30 mg/kg) directly induced lung thrombosis formation, white arrows in **F** indicate thrombosis in lung, scale bar: 100 μm. Data are mean ± SD of at least three independent experiments. *p < 0.05, **p < 0.01

As LL-37 was correlated with TT and fibrinogen, the effects of LL-37 on coagulation factor activity were determined. Based on the chromogenic substrate assay, LL-37 enhanced the activity of thrombin (Fig. 4A) and FXa (Fig. 4B) in a dose-dependent manner. SPR was conducted to analyze the binding capacity of LL-37 with thrombin and FXa. As illustrated in Fig. S1A, S1B, LL-37 could bind to thrombin and FXa, with K_D_ values of 2.64 × 10^–6^ M and 8.47 × 10^–7^ M, respectively. We also measured the effects of LL-37 on thrombin and FXa to their physiological substrates. After the reactions of thrombin or FXa with fibrinogen or prothrombin in the presence of cathelicidin peptides (0–50 μg/ml), the samples were separated by SDS-PAGE and analyzed using Coomassie Blue. Similar to the chromogenic substrate assay, LL-37 enhanced the activity of thrombin and FXa against their physiological substrates, fibrinogen (Fig. 4C) and prothrombin (Fig. 4D), respectively.

Given the effect of LL-37 on thrombin and FXa, we further investigated the effects of cathelicidin on thrombosis formation in vivo. As seen in Fig. 4E, LL-37 significantly promoted thrombosis formation and shortened the time of arterial occlusion in the FeCl_3_-induced carotid artery thrombosis mouse model. LL-37 administration also induced lung thrombosis directly (Fig. 4G).

### Cramp promotes thrombosis formation through promotion of coagulation factor activity

**Fig. 5:**
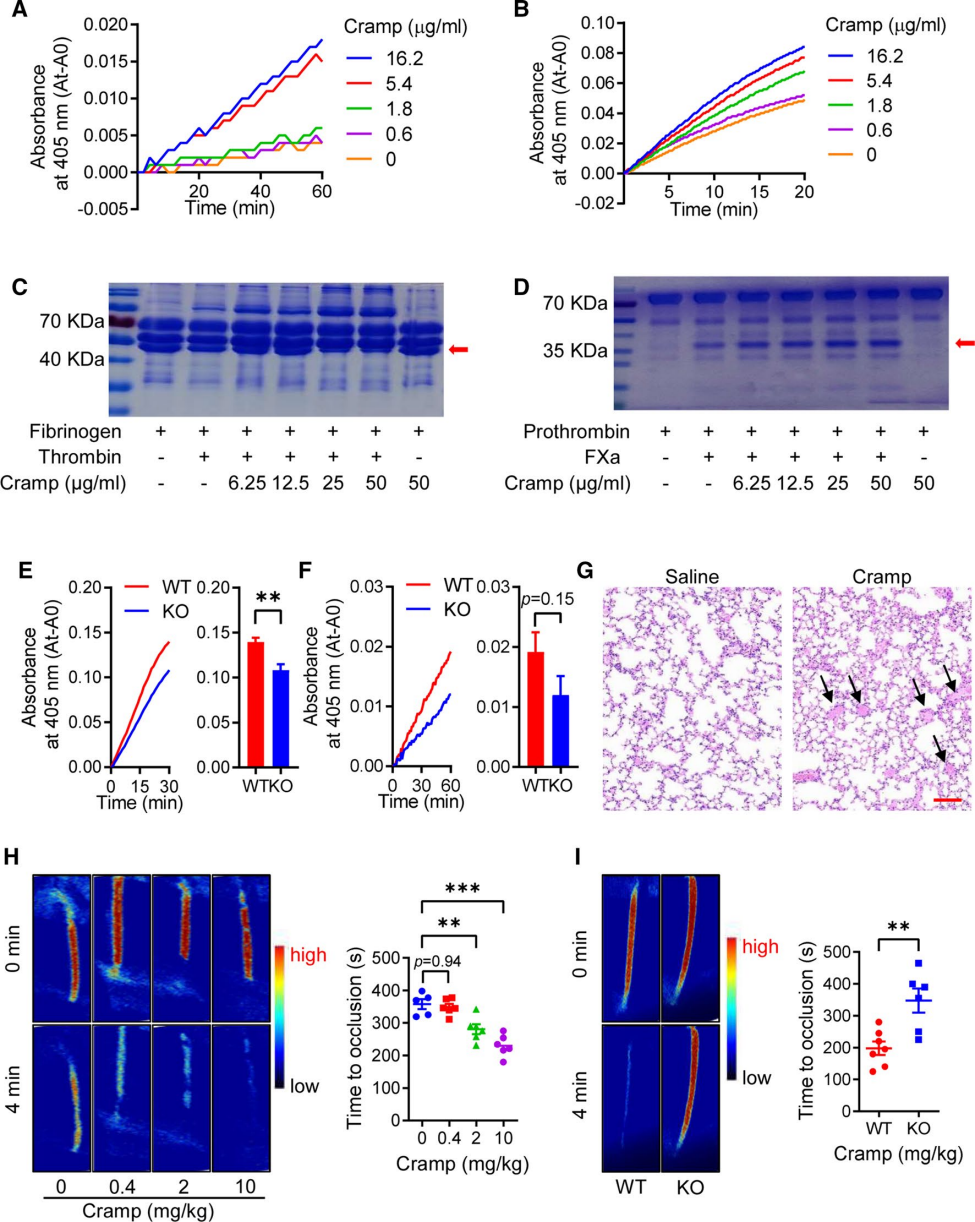
Effects of Cramp on thrombosis formation through potentiation of coagulation factor activity. Cramp potentiated activity of thrombin (**A**, **C**) and FXa (**B**, **D**) on their chromogenic (**A**, **B**) and physiological substrates (**C**, **D**). (**E**, **F**) C57BL/6 wild-type (WT) mouse plasma and *Cramp*^*−/−*^ mouse plasma were added to chromogenic substrates of coagulation factors (**E**: thrombin, **F**: FXa), and absorbance at 405 nm was measured immediately for 30 min, 60 min, respectively. (**G**) Cramp (30 mg/kg) directly induced lung thrombosis formation, black arrows in Figure **G** indicated thrombosis in lung, scale bar: 100 μm. (**H**) Cramp administration (0.4–10 mg/kg) induced thrombosis formation and shortened time of arterial occlusion in FeCl_3_-induced carotid artery thrombosis mouse model. Representative images of carotid artery blood flow at 0 and 4 min (left), and statistical analysis of vascular occlusion time (right). (**I**) *Cramp* deletion inhibited FeCl_3_-induced carotid artery thrombosis formation and extended time of arterial occlusion. Representative images of carotid artery blood flow at 0 and 4 min (left), and the statistical analysis of vascular occlusion time (right). Data are mean ± SD of at least three independent experiments. *p < 0.05, **p < 0.01, ***p < 0.001

To confirm the effects of LL-37 on thrombosis formation through activation of coagulation factors, we detected the effects of Cramp (LL-37 homolog from *Mus musculus*) on coagulation factor activation. Similar to the LL-37 results, the chromogenic substrate assay showed that Cramp significantly enhanced the enzymatic activity of thrombin (Fig. 5A) and FXa (Fig. 5B) in a dose-dependent manner. SPR analysis also indicated interactions of Cramp with thrombin (Fig. S1C) and FXa (Fig. S1D), with K_D_ values of 6.78 × 10^–6^ M and 3.17 × 10^–4^ M, respectively. Based on the K_D_ results, the binding capacity of LL-37 with coagulation factors was higher than that of Cramp. The results of thrombin and FXa on their natural substrates (fibrinogen and prothrombin) confirmed that Cramp promoted the enzymatic activity of thrombin (Fig. 5C) and FXa (Fig. 5D).

Furthermore, plasma from *Cramp*^*−/−*^ mice showed weaker thrombin (Fig. 5E) and FXa activity (Fig. 5F) on chromogenic substrates than plasma from wild-type C57BL/6 mice, suggesting that Cramp deletion attenuated thrombin and FXa activity.

Similar to the LL-37 results, Cramp administration significantly enhanced lung thrombosis in acute pulmonary thromboembolism mouse model (Fig. 5G) and shortened the time of arterial occlusion in the FeCl_3_-induced carotid artery thrombosis mouse model (Fig. 5H). Furthermore, Cramp deletion was resistant to arterial occlusion in the FeCl_3_-induced carotid artery thrombosis mouse model (Fig. 5I).

### LL-37 and Cramp show similar effects on PT and APTT in COVID-19 patients.

LL-37 and Cramp enhanced coagulation factor (thrombin and FXa) activity, which should shorten PT and APTT. However, LL-37 had no effect on PT (Fig. S2A) and prolonged APTT (Fig. S2B) at higher concentrations. Somewhat inconsistent with LL-37, Cramp prolonged PT (Fig. S2C) and APTT (Fig. S2D) in a dose-dependent manner. Thus, these findings were contradictory to expectation, but were consistent with the PT and APTT values found in COVID-19 patients.

Basic histone, which can induce hypercoagulation, prolongs PT and APTT by binding with phospholipids [41], suggesting that basic LL-37 or Cramp may prolong PT and APTT through interaction with phospholipids. Indeed, based on the ELISA results, LL-37 and Cramp could bind with cardiolipin (Fig. S3A).Furthermore, we detected the effects of pre-incubation with cardiolipin on cathelicidin peptide-induced PT/APTT prolongation. LL-37 induced PT prolongation tendency at concentration of 25 μM, which could be inhibited by pre-incubation with cardiolipin (Fig. S3B). In addition, LL-37-induced APTT prolongation was significantly inhibited by cardiolipin pre-incubation (Fig. S3C). Similar to LL-37, pre-incubation with cardiolipin significantly abolished Cramp-induced PT (Fig. S3D) and APTT (Fig. S3E) prolongation. From these results, PT/APTT prolongation induced by LL-37 or Cramp may be caused by interactions with phospholipids, which can inhibited by the pre-incubation with cardiolipin.

## Discussion

**Fig. 6:**
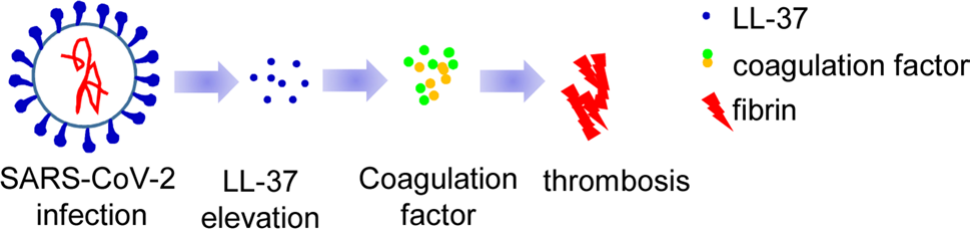
Schematic diagram of role of LL-37 in hypercoagulation in COVID-19 patients. SARS-CoV-2 infection increased LL-37, which induced thrombosis formation through potentiation of coagulation factor activity

To the best of our knowledge, this is the first study to report on the correlation between elevated LL-37 levels and hypercoagulation in COVID-19 patients. LL-37 was upregulated by SARS-CoV-2 infection to cause elevated concentration in the plasma of COVID-19 patients and showed the ability to directly activate coagulation factors. The upregulation of LL-37 was associated with clinical hypercoagulation manifestations induced by SARS-CoV-2 infection and likely contributes to the hypercoagulation frequently observed in COVID-19 patients (Fig. 6).

Hypercoagulability has been reported as a central pathological feature and clinical complication in COVID-19 [10]. It is likely that multiple systems contribute to thrombosis in COVID-19 patients, such as coagulation activation, platelet activation, hypofibrinolysis, endothelial cell dysfunction, inflammation, NETs, and complement [42]. Platelet activation and platelet-monocyte aggregate formation trigger tissue factor expression in patients with severe COVID-19 [43]. NETs are also known to contribute to immunothrombosis in COVID-19 acute respiratory distress syndrome [27]. Here, we showed that LL-37 induced hypercoagulability through enhancement of coagulation factor activity. LL-37 has also been found to induce endothelial cell dysfunction, inflammation, NETs formation, platelet activation, which may promote thrombosis in COVID-19.

The hCAP18 protein contains a conserved cathelin-like domain and a highly variable C-terminal peptide (LL-37). The cathelin-like domain of the cathelicidin is classified into the same superfamily as cystatins, the cysteine protease inhibitors, and the cathelin-like domain of hCAP18 can inhibit cathepsin L activity [44]. Although few studies have explored the function of the cathelicidin C-terminal peptide on protease activity, a recent study on cathelicidin-MH (cath-MH) from the skin of Microhyla heymonsivogt frog was found to suppress coagulation by affecting enzymatic activities [45], inconsistent with the effects of LL-37. Through sequence alignment analysis, LL-37 displays low sequence identity with cath-MH and lacks the loop formed by the intramolecular disulfide bond [45], which may explain their discrepancy in coagulation factor activity.

Disseminated intravascular coagulopathy (DIC) has been reported in COVID-19 patients [46]. In addition, consistent with classic DIC caused by bacterial sepsis, prolonged APTT, thrombocytopenia, elevated D-dimer, and multi-organ microangiopathic thrombosis have also been found in COVID-19 patients. The prolongation of APTT is difficult to explain, but our results suggest it may be induced by elevated LL-37. Notably, LL-37 activates the thrombin and FXa coagulation factors, leading to hypercoagulability, with the binding of phospholipids likely prolonging APTT.

As a major family of antimicrobial peptides, cathelicidin peptides are expressed over a broad range of sites during infection and inflammation, and are primarily generated by neutrophils and epithelial cells [47]. Elevated cathelicidins form part of the body’s defense against pathogens, with the antiviral activity of cathelicidin peptides reported in many viruses e.g., human immunodeficiency virus (HIV)-1 [48], influenza A virus (IAV) [31, 49], respiratory syncytial virus (RSV) [47], rhinovirus (HRV) [50], vaccinia virus (VACV) [51], herpes simplex virus (HSV) [52], zika virus (ZIKV) [53], dengue virus (DENV) [54] and hepatitis C virus (HCV) [55]. Recently, research reported the inhibition of LL-37 on SARS-CoV-2 infection using biochemical and pseudovirus entry assays [30]. While, the direct effects of LL-37 on SARS-CoV-2 remain unclear. Here we found LL-37 induced hypercoagulation through the potentiation of coagulation factor activities, which was consistent with the clinical symptoms of COVID-19 patients. Moreover, elevated LL-37 has been found to activate platelets. Therefore, although the elevation of LL-37 during SARS-CoV-2 infection may be a protective mechanism of the innate immune system, the increase in LL-37 may also aggravate disease progress by inducing thrombosis, which may explain the controversy of vitamin D (inducer of LL-37 production) treatment in COVID-19 [56].

In addition to its antimicrobial activity, LL-37 also exhibits various biological effects, such as regulation of inflammation, cell proliferation and apoptosis. However, overexpression of LL-37 or LL-37 complexes with other molecules may contribute to progression of diseases. For example, the LL-37-DNA/RNA complex can aggravate psoriasis [34, 57], atherosclerosis [35, 58], ulcerative colitis [36], sepsis [59], thrombosis [37], and chronic obstructive pulmonary disease [60] through the induction of inflammation. Cytokine storms are another clinical marker of SARS-CoV-2, which induce higher morbidity and mortality in COVID-19 patients [61]. Circulating cell-free DNA [62], cell-free mitochondrial DNA [63], and cell-free microbial DNA [64] are increased in SARS-CoV-2 infection and are associated with disease severity and mortality in COVID-19 patients. Therefore, elevated LL-37 may induce the inflammation via interactions with increased cell-free DNA of COVID-19 patients, thereby exacerbating disease process.

In conclusion, we observed a close correlation between LL-37 and the hypercoagulation frequently observed in COVID-19 patients. The level of LL-37 was increased in the plasma of COVID-19 patients with the induction of SARS-CoV-2 spike protein. Elevated LL-37 may contribute to thrombosis via potentiation of coagulation factor activity. As the results of our research, although LL-37 has been found to perturb SARS-CoV-2 infection, it is not suitable for the treatment of SARS-CoV-2 infection, especially for patients in hypercoagulability.

### Supplementary Information

Below is the link to the electronic supplementary material.

Supplementary file1 (DOCX 596 kb)

## Data Availability

Data are available on request from the corresponding author.
