# Supplementary material for: Role of LL-37 in thrombotic complications in patients with COVID-19
Source: Cell Mol Life Sci. 2022 May 21;79(6):309. doi: 10.1007/s00018-022-04309-y (PMC9123294; doi:10.1007/s00018-022-04309-y)
Supplement: Supplementary file 1 — Supplementary file1 (DOCX 596 kb) [file 18_2022_4309_MOESM1_ESM.docx]

**Supplementary Materials**

**Supplementary Figures**

**
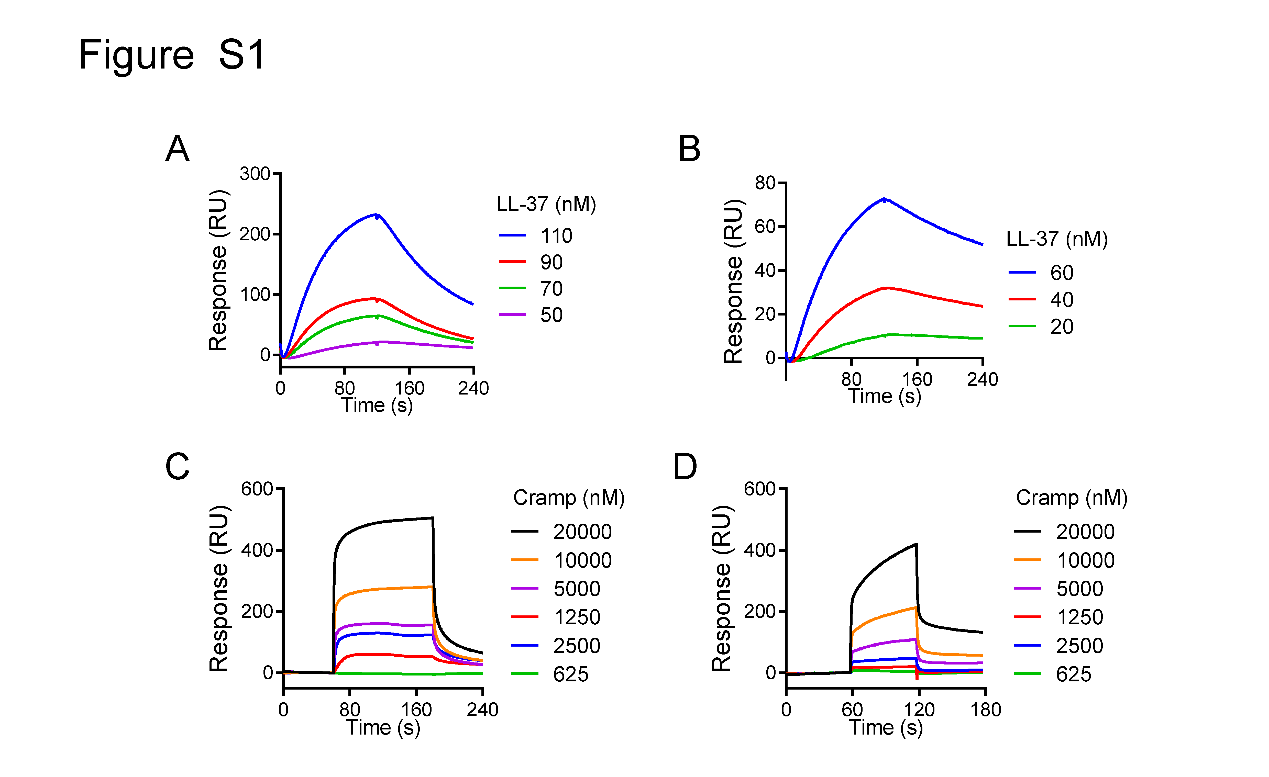
**

**Fig. S1.** Interactions between cathelicidin antimicrobial peptides with thrombin and FXa. (A, B) Surface plasmon resonance (SPR) analysis of interaction between LL-37 and thrombin (A) and FXa (B) on sensor CM-5 chip. LL-37 could bind to thrombin and FXa, with equilibrium dissociation constants (K_D_) of 2.64 × 10^-6^ M and 8.47 × 10^-7^ M, respectively. (C, D) SPR analysis of interaction between Cramp and thrombin (C) and FXa (D) on sensor CM-5 chip. Cramp could bind to thrombin and FXa with K_D_ values of 6.78 × 10^-6^ M and 3.17 × 10^-4^ M, respectively.

**
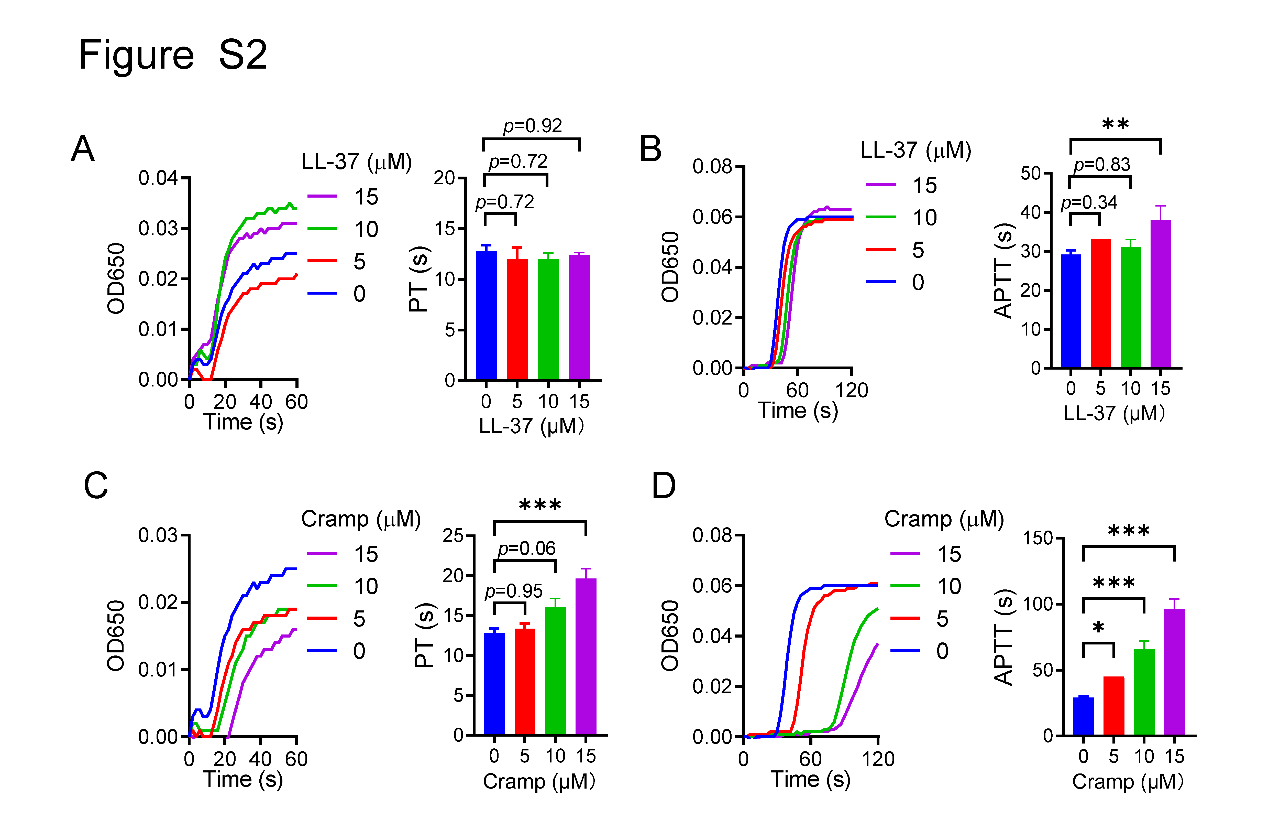
**

**Fig. S2.** Effects of cathelicidin peptides on PT and APTT. (A) LL-37 showed no effect on PT. (B) LL-37 prolonged APTT at the concentration of 15 μM. (C) Cramp prolonged PT in a dose-dependent manner. (D) Cramp prolonged APTT in a dose-dependent manner. Data are mean ± SD of at least three independent experiments. **p* < 0.05, ***p* < 0.01, ****p* < 0.001.

**
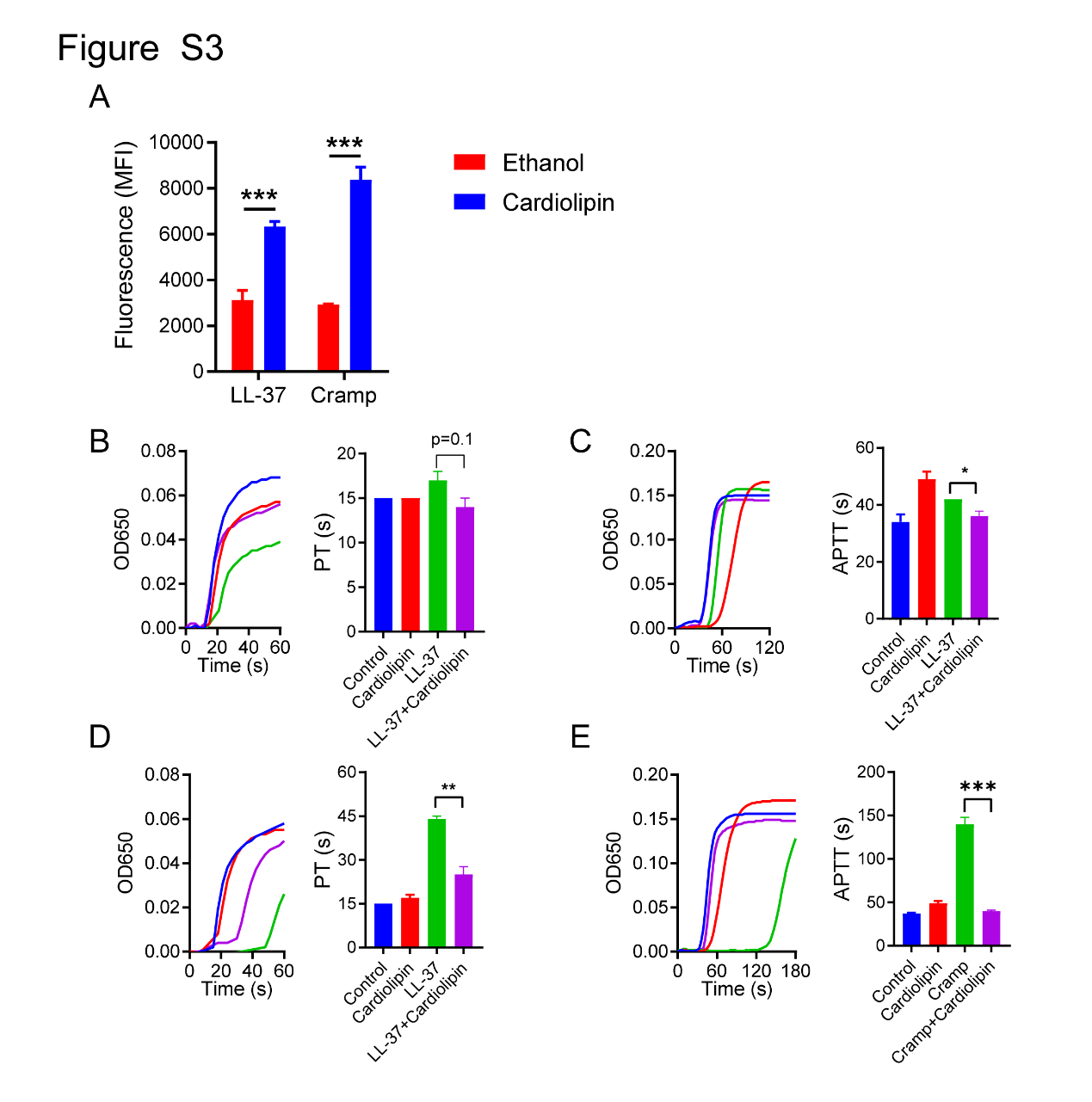
Fig. S3.** Cathelicidin peptides prolonged PT/APTT by binding to phospholipids. (A) Cardiolipin (50 μg/ml) was bound to the 96- well white platelet, with FITC-labelled LL-37 or Cramp (10 μg/ml) added to detect their interactions. (B) Pre-incubation with cardiolipin (100 μg/ml) inhibited LL-37 (25 μM)-induced PT prolongation tendency. (C) Pre-incubation with cardiolipin (100 μg/ml) significantly inhibited LL-37 (25 μM)-induced APTT prolongation. (D) Pre-incubation with cardiolipin (100 μg/ml) significantly abolished Cramp (25 μM)-induced PT prolongation. (C) Pre-incubation with cardiolipin (100 μg/ml) significantly abolished Cramp (25 μM)-induced APTT prolongation. Data are presented as the mean ± SD of independent experiments (n ≥ 3). **p* < 0.05, ***p* < 0.01, ****p* < 0.001.
